# Surveillance of Russell body inflammation of the digestive tract: a case report and review of literature

**DOI:** 10.1186/s13000-022-01242-3

**Published:** 2022-08-25

**Authors:** Shuai Luo, Xiang Huang, Yao Li, Jinjing Wang

**Affiliations:** Department of Pathology, Affiliated Hospital of Zunyi Medical University, Zunyi City, Guizhou Province P.R. China

**Keywords:** Russell body, Mott cells, Inflammation, Pathology, Diagnosis

## Abstract

**Introduction:**

Russell body inflammation of the digestive tract (RBIDT) is a rare chronic inflammation of the digestive tract mucosa that commonly presents as Russell body gastritis (RBG). This disease is usually associated with *Helicobacter pylori* (HP) infection. However, it can also occur in individuals without HP infection and with specific immune profiles, as seen in HIV and hepatitis C infections. The aetiology and pathogenesis of this disease remain controversial. Given the rarity of the disease and the diversity of the immunophenotypes, there is a high probability of misdiagnosis.

**Case presentation:**

A male patient with RBG and HP infection was included in this study. The case of RBG with an unusual morphology of Mott cells that mimics stamped ring cells.Endoscopy performed during the follow-up revealed regression of the lesion 1 week after anti-HP treatment.

**Conclusions:**

A case of RBG with a high likelihood of misdiagnosis of signet ring cell carcinoma (SRC) has been reported in this study along with a review of the relevant literature and an overview of RBIDT.

## Introduction

RBG was first reported by Tazawa et al. [1] as an incidental finding in 1998 and is considered a benign inflammatory change. According to statistical data, HP infection is present in approximately two-thirds of the patients with RBG. With an increase in the number of reported cases of RBG, such inflammatory lesions have been found to occur in almost the entire digestive tract (from the oesophagus to the rectum) and in the heart. Therefore, we have described a case of RBG and an overview of RBIDT in this study.

### Case presentation

**Fig. 1:**
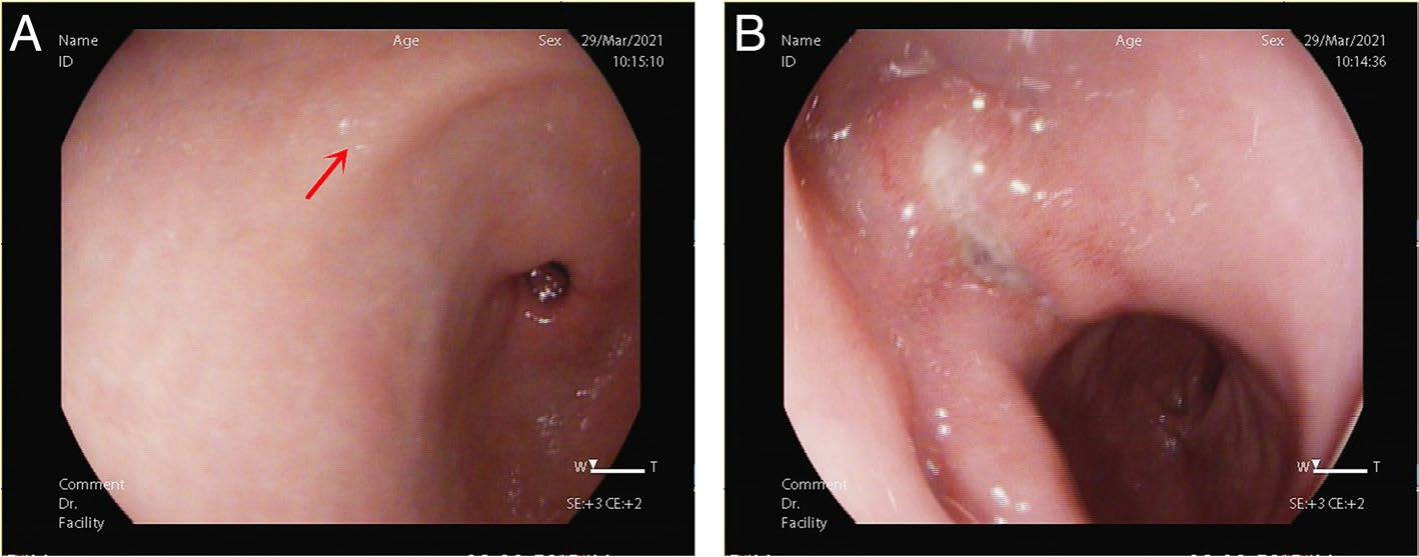
Gastroscopy revealed congestion and oedema in the mucosa of the gastric antrum, with reddish-white-coloured (predominantly white) and punctate erosions (**A**, The red arrow is the biopsy forceps extraction point). A 5 × 10-mm ulcer was observed in the fovea of the duodenal bulb with surrounding mucosal congestion, oedema and smudged moss (**B**)

A 50-year-old man presented before 2 years with a complaint of change in stool property (dry or thin and unformed with occasional tenesmus) and was diagnosed with irritable bowel syndrome owing to recurrent symptoms. Recently, he was admitted to our hospital with a dull pain in the upper and middle abdomen with occasional hiccoughs and sour regurgitation. Physical examination revealed pressing pain in the epigastric region. The C^14^ breath test result was positive for HP. Gastroscopy revealed congestion and oedema in the mucosa of the gastric antrum, with reddish-white-coloured (predominantly white) and punctate erosions (Fig. 1A). A 5 × 10-mm ulcer was observed in the fovea of the duodenal bulb with surrounding mucosal congestion, oedema and smudged moss (Fig. 1B). The mucosa of the whitened area of the gastric antrum was acquired with medical forceps for biopsy.

**Fig. 2:**
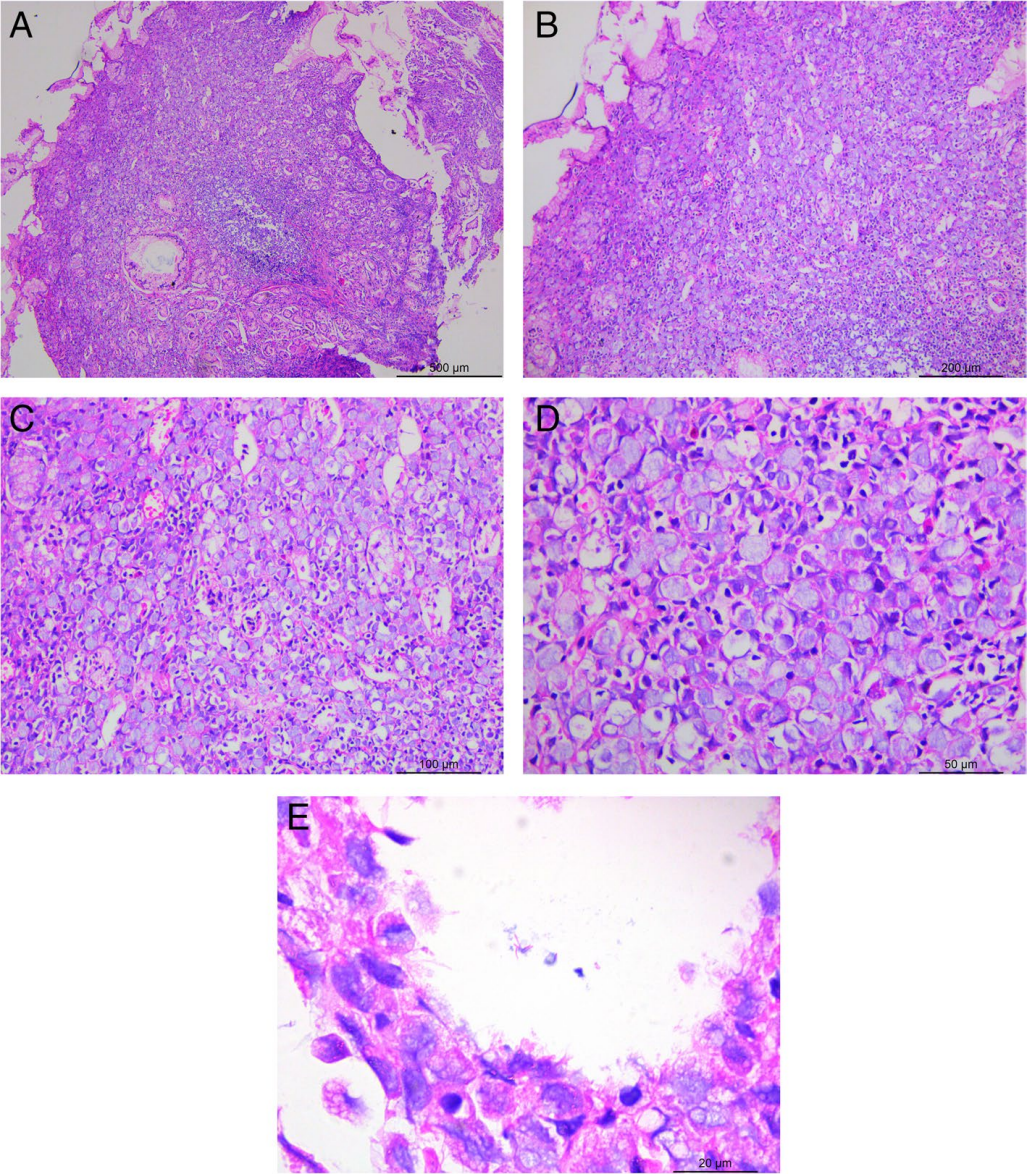
Microscopically, a large number of cytoplasm-rich, nucleus-deviating, ring-like cells, 5–13 μm in diameter, with basophilic mucus-like material in the cytoplasm, are seen between the glands of the lamina propria, and the cells are distributed in foci or sheets, like grapes (**A**:H&E stain ×50, **B**:H&E ×100, **C**: H&E stain ×200, **D**:H&E stain ×400). Several short, thin rods of H. pylori were seen in the gastric mucosal space(**E**: H&E stain ×1000)

**Fig. 3:**
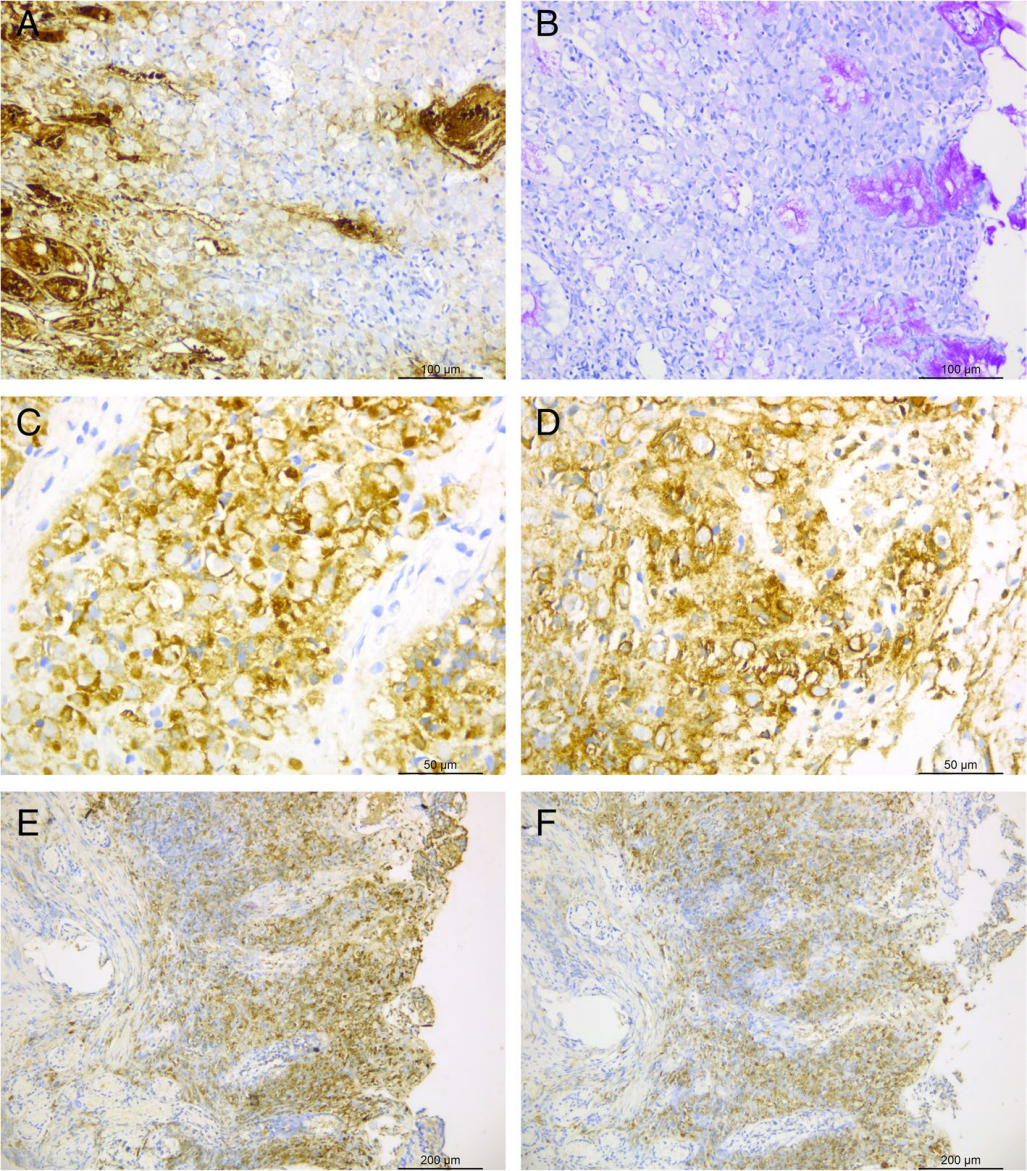
Immunohistochemical stains in mimics signet ring cells negative (initial biopsy). **A**: CK(-); **B**: PAS(-); **C**: CD79a(+++); **D**: CD138 (++); **E**: Kappa (κ) (++); F:Lambda (λ) (++)

Histopathological evaluation did not reveal intestinal metaplasia of the epithelial cells and mitosis or dysplasia in the mucosa of the gastric antrum. However, a chronic inflammatory cell infiltration was observed in the mesenchyme, along with a large number of cytoplasm-rich, nuclear-deviated mimics signet ring cells with a diameter of 5–13 μm between the glands of the lamina propria. A basophilic mucus-like substance was observed in the cytoplasm, and the cells were distributed in a focal or lamellar manner like grapes (Fig. 2A-D). Short, thin rods of H. pylori were seen in the small gastric pits and glandular cavities on the surface of the gastric mucosa(Fig. 2E).Immunohistochemical analysis revealed that the mimics signet ring cells were CK (-) (Fig. 3A), PAS (-) and D-PAS (-) (Fig. 3B), which excluded the possibility of mimics signet ring cell carcinoma (SRC) of epithelial origin. LCA (+++), MUM1 (+++), CD79a (+++) (Fig. 3C) and CD138 (++) (Fig. 3D) confirmed a plasma cell nature. Kappa (κ) (+++) (Fig. 3E), Lambda (λ) (++) (Fig. 3F) and molecular tests revealed polyclonal rearrangements of B cells, suggesting a polyclonal phenotype. The negative expressions of CD20, Bc1-2, Bc1-6, CD163, CDX2, CEA, CK20, CK7, CD5, ALK, CD3, CD56, cyclin D1 and SOX-11 ruled out other suspected malignancies, whereas Ki-67 (approximately 1%+) suggested a benign lesion. Therefore, the diagnosis favoured RBG with polyclonal plasma cell hyperplasia.

**Fig. 4:**
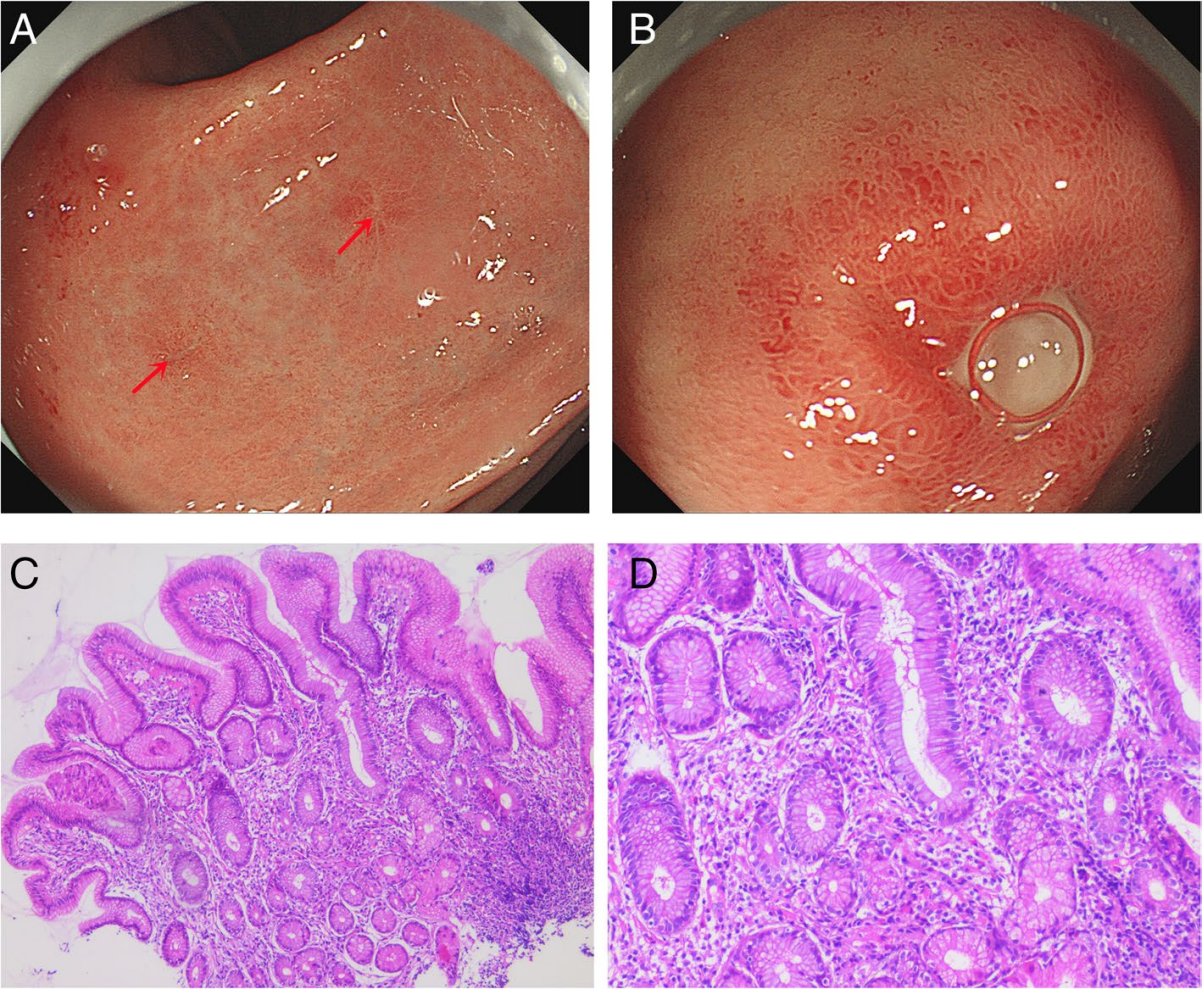
**A:** Two scattered erosions seen on endoscopy after 1 week of anti-HP treatment,the red arrow is the biopsy forceps extraction point; **B**: a smaller bulbous ulcer on endoscopy. **C **(H&E ×50) and **D **(H&E ×100): Pathological findings on gastroscopic forceps biopsy of tissue

After 1 week of anti-HP treatment, an endoscopy performed during the follow-up revealed scattered foci of erosion in the anterior wall of the gastric antrum (Fig. 4A), with a smaller bulbous ulcer measuring approximately 2 × 3 mm (Fig. 4B). A biopsy of the mucosa from the erosion zone of the gastric antrum revealed interstitial oedema, lymphocytic and neutrophilic infiltration, atrophy of the proper gastric glands and complete disappearance of the mimics signet ring cell (Fig. 4C and D).

**Fig. 5:**
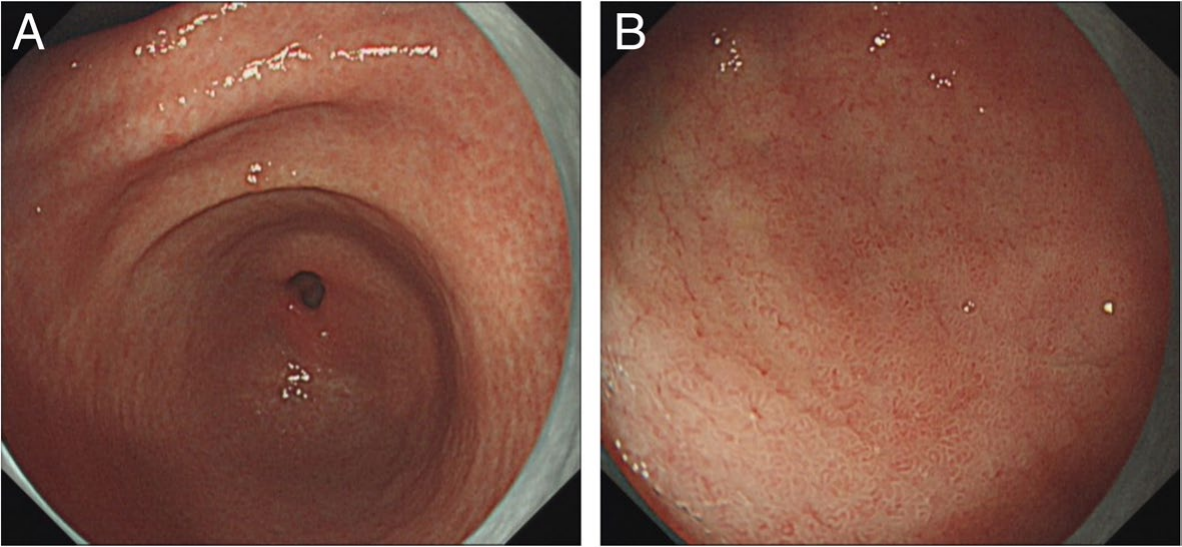
Endoscopy after 7 months of anti-HP treatment: there was no significant abnormality in the mucosa of the gastric sinus (**A**), ulcerative scars were visible in the mucosa of the bulb (**B**)

After regular anti-HP treatment, the gastroscopy was repeated 7 months later: there was no erosion in the mucosa of the gastric sinus(Fig. 5A), ulcerative scars were visible in the mucosa of the bulb(Fig. 5B), and the C^14^ breath test result was negative for HP,therefore, the endoscopist did not clamp the gastric mucosal tissue for pathological biopsy .

The patient is currently in good condition with no discomfort such as acid reflux and belching.

## Discussion

RB, first reported by Russell [2] in 1890, is an eosinophilic corpuscle formed by the disturbed secretion and accumulation of immunoglobulins (IGs) within the swollen rough endoplasmic reticulum. Plasma cells containing RBs are known as Mott cells. RBG was first described by Tazawa and Tsutsumi [1] in 1998 as microscopic chronic inflammatory changes dominated by massive Mott cell infiltration in the lamina propria of the gastric mucosa. With increased recognition and reporting of this disease, RBs have been found to occur in almost the entire digestive tract. We have presented an overview of RBIDT in this study to understand the phenomenon better.

### Relevant literature search

The Pubmed database was searched for literature using the search term “Russell body”, and case reports of inflammatory lesions in the gastrointestinal tract of RB were used as inclusion criteria, resulting in 51 articles from 1998 to the present, plus 76 cases in this case.

### Analysis of clinicopathological features

#### Prevalent population and site

**Fig. 6:**
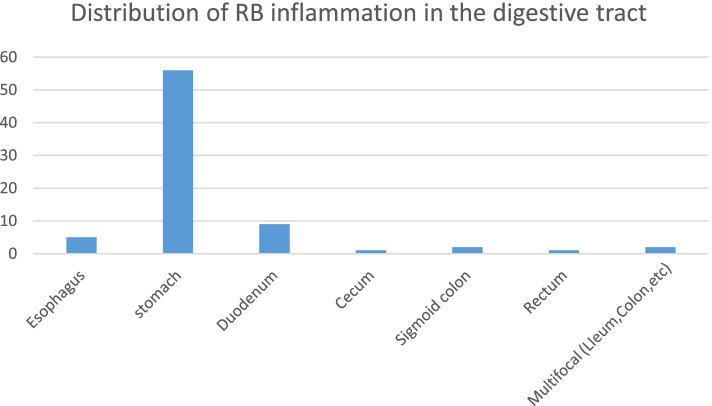
Distribution of Russell body inflammation in the digestive tract: in all cases from the literature

There were 76 cases of RBIDT, which occurred in middle-aged and elderly males, with a wide range of age (18–88 years) and a mean age of 63 years, 1.71 times more males than females (48/28). 5 of the 76 cases were in the oesophagus, 56 in the stomach, 9 in the duodenum, 1 in the cecum, 2 in the sigmoid colon, 1 in the rectum and 2 in multifocal (Fig. 6).

#### Clinical symptoms and endoscopic findings

Patients mostly present with non-specific gastrointestinal symptoms (abdominal pain, dyspepsia and nausea) or may be asymptomatic and found incidentally on physical examination. Endoscopic features are also non-specific, such as mucosal erythema, whitening, oedema, erosions and ulcers, sometimes as raised nodules.

#### Russell body Barrett’s esophagus (RBBE) (Table 1)

**Table 1:** Clinical and pathologic findings of previously published cases of RBBE

Case	Study		Reported Number of Cases	Age(yr)	sex	Location	*Helicobacter pylori* Infection	Ig light chain of Mott cells	Other Conditions	Follow up
1	Rubio et al. [3]	2005	1	88	M	Esophagus	NS	Polyclonal	Barrett’s esophagus	NS
2	Bhaijee et al. [4]	2012	1	71	M	Esophagus	NS	Polyclonal	Barrett’s esophagus	NS
3	Rangan et al. [5]	2016	1	80	M	Esophagus	No	Polyclonal	Barrett’s esophagus	NS
4	Arshi et al. [6]	2020	1	41	M	Esophagus,gastric cardia	NS	Polyclonal	Barrett’s esophagus,Candida esophagitis	NS
5	Dhorajiya et al. [7]	2020	1	82	M	Esophagus	NS	Polyclonal	Barrett’s esophagus	NS

Five cases, all male, with Barrett’s esophagus as the underlying disease and one case occurring in Barrett’s esophagus and cardia gastric mucosa, so it can be assumed that the disease only occurs in the lamina propria of the overlying columnar epithelium, and that there may be Candida infection in the esophagus, but no HP infection was reported, so it was not considered to be HP-related.

#### Russell body duodenitis(RBD) (Table 2)

**Table 2:** Clinical and pathologic findings of previously published cases of RBD

Case	Study		Reported Number of Cases	Age(yr)	sex	Location	*Helicobacter pylori* Infection	Ig light chain of Mott cells	Other Conditions	Follow up
1	Savage et al. [8]	2011	1	55	M	Duodenum	No	Polyclonal	HIV+, Lymphoma	NS
2	Paniz Mondolf et al. [9]	2012	1	69	F	Duodenum	No	Polyclonal	Autoimmune Disease	NS
3	Takahashi et al. [10]	2013	1	77	M	Duodenum	No	Polyclonal	Urothelial carcinoma	NS
4	Chen et al. [11]	2013	1	59	F	Duodenum	Yes	Polyclonal	Diabetes, Hypertension、COPD	NS
5	Zhang et al. [12]	2014	1	76	M	Duodenum	Yes	Monoclonal (λ)	CP	NS
6	Munday et al. [13]	2015	1	78	F	Duodenum	No	Monoclonal (κ)	Heart failure, COPD	NS
7	Goto et al. [14]	2016	1	64	M	Duodenum	No	Polyclonal	Duodenal ulcer,Pulmonary cryptococcosis	NS
8	Dissanayake et al. [15]	2018	1	82	F	Duodenum	No	Monoclonal (κ)	Sjogren’s syndrome、SLE	NS
9	Altindag et al. [16]	2019	1	68	M	Duodenum	No	Polyclonal	NS	NS

Nine cases, five men and four women, two with HP infection and three with Mott cells exhibiting a monoclonal immunophenotype. Patients may have chronic enteritis, colonic polyps and immune dysfunctional diseases such as autoimmune diseases, HIV and uroepithelial carcinoma.

#### Russell body coloproctitis(RBCR) (Table 3)

**Table 3:** Clinical and pathologic findings of previously published cases of RBCR

Case	Study			Reported Number of Cases	Age(yr)	sex	Location		*Helicobacter pylori* Infection	Ig light chain of Mott cells		Other Conditions	Follow up	
1	Brink et al. [17]		1999	1	53	F	Rectum		No	Monoclonal (κ)		Rectal polyp	NS	
2	Muthukumarana et al. [18]		2015	1	44	F	Multifocal(Stomach,Duodenum, Lleum,Colon)		No	Polyclonal		Diabetes, post pancreatic and left kidney transplant,	NS	
3	Coates et al. [19]		2017	1	62	M	Sigmoid colon		No	Polyclonal		Hypertension, CP	NS	
4	Xu et al. [20]		2019	1	18	M	Multifocal(Jejunum, Lleum,Colon,Rectum)		No	Monoclonal (λ)		Peutz-Jeghers syndrome	NS	
5	Al-Rawaf et al. [21]		2021	1	78	M	Cecum		No	Polyclonal		Liver cirrhosis, Chronic kidney disease, Urothelial carcinoma, History of NSAID use	NS	
6	Tan et al. [22]		2021	1	77	M	Sigmoid colon		No	Monoclonal (λ)		CP	NS	

Six cases, four males and two females, occurring in the cecum, sigmoid colon, rectum or multiple sites; Mott cell manifestations 2 monoclonal λ-chain immunophenotypes and 1 monoclonal κ-chain immunophenotype, patients with underlying disease similar to RB duodenitis, may have inflammation, polyps and immune dysfunctional disease (diabetes, organ transplantation, NSAID drugs, etc.).

#### RBG (Table 4)

RBG is the most common type of RBIDT. A total of 56 cases (male: female = 1.6:1 [34/22]; average age, 62 years) were included in this study; of which, 66% (37) had HP infection. In addition, 13 Mott cells exhibited a monoclonal κ-chain immunophenotype.

In this study, RBG was categorised as HP-positive and HP-negative based on the presence or absence of HP infection.

**Table 4:** Clinical and pathologic findings of previously published cases of RBG

Case		Study		Reported Number of Cases	Age(yr)	sex		Location	*Helicobacter pylori* Infection	Ig light chain of Mott cells	Other Conditions			Follow up
1		Tazawa et al. [1]	1998	1	53	M		Stomach	Yes	Polyclonal	Alcoholic cirrhosis of the liver			almost absent RB after radical treatment of HP
2		Erbersdobler et al. [23]	2004	1	80	F		Stomach	No	Polyclonal	Candida esophagitis, history of psychosis			NS
3		Ensari et al. [24]	2005	1	70	M		Stomach	Yes	Polyclonal	Hypertension			NS
4		Drut et al. [25]	2006	1	34	M		Stomach	No	Polyclonal	HIV+, Alcohol abuse			NS
5		Wolkersdörfer et al. [26]	2006	1	54	M		Stomach	Yes	Polyclonal	MGUS			NS
6		Paik et al. [27]	2006	2	47	F		Stomach	Yes	Polyclonal	CG			almost absent RB after radical treatment of HP
7					35	F		Stomach	Yes	Polyclonal	CG			almost absent RB after radical treatment of HP
8		Pizzolitto et al. [28]	2007	1	60	F		Stomach	Yes	Polyclonal,PAS(+)	CG			almost absent RB after radical treatment of HP
9		Eum et al. [29]	2007	1	48	M		Stomach	Yes	NS	CP			NS
10		Licci et al. [30]	2009	1	59	M		Stomach	Yes	Polyclonal	HIV+			almost absent RB after radical treatment of HP
11		Habib et al. [31]	2010	1	75	M		Stomach	No	Polyclonal	Hyperlipidemia,Rhabdomyolysis			NS
12		Shinozaki et al. [32]	2010	2	74	M		Stomach	Yes	Polyclonal	EBVAGC			NS
13					29	F		Stomach	Yes	Polyclonal	EBVAGC			NS
14		Del Gobbo et al. [33]	2011	1	78	F		Stomach	No	Polyclonal	CG			NS
15		Wolf et al. [34]	2011	1	67	M		Stomach	Yes	NS	Signet-ring cell carcinoma			NS
16		Yoon et al. [35]	2012	2	57	M		Stomach	Yes	Polyclonal	GP,CP			almost absent RB after radical treatment of HP
17					43	M		Stomach	Yes	Polyclonal	CG			almost absent RB after radical treatment of HP
18		Bhalla et al. [36]	2012	1	82	M		Stomach	No	Polyclonal	HIV+			NS
19		Coyne et al. [37]	2012	1	49	M		Stomach	No	Monoclonal (κ)	Hepatitis c			NS
20		Karabagli et al. [38]	2012	1	60	M		Stomach	Yes	Polyclonal	CG			NS
21		Choi et al. [39]	2012	1	55	M		Stomach	Yes	NS	GC			NS
22		Miura et al. [40]	2012	1	63	F		Stomach	Yes	Monoclonal (κ)	CG,Hyperlipidemia			NS
23		Araki et al. [41]	2013	1	74	F		Stomach	Yes	Monoclonal (κ)	Alzheimer’s disease, Stomach ulcers			NS
24		Zhang et al. [12]	2014	9	78	F		Stomach	No	Monoclonal (κ)	NS			Clinical followup evaluations were uneventful
25					77	F		Stomach	Yes	Monoclonal (κ)	NS			NS
26					77	F		Stomach	Yes	Monoclonal (κ)	CP			NS
27					56	M		Stomach	Yes	Monoclonal (κ)	CP			NS
28					76	M		Stomach	Yes	Monoclonal (κ)	NS			NS
29					50	M		Stomach	Yes	Monoclonal (κ)	NS			NS
30					28	M		Stomach	No	Monoclonal (κ)	NS			NS
31					24	F		Stomach	No	Monoclonal (κ)	NS			NS
32					66	M		Stomach	No	NS	NS			NS
33		Antunes et al. [42]	2016	1	79	F		Stomach	No	NS	GERD			NS
34		Nishimura et al. [43]	2016	1	64	F		Stomach	Yes	Polyclonal	Bronchiectasis			almost absent RB after radical treatment of HP
35		Imai et al. [44]	2016	1	64	M		Stomach	No	Polyclonal	Eosinophilia			NS
36		Zhang et al. [45]	2016	1	69	M		Stomach	Yes	Monoclonal (κ)	Hypertension			NS
37		Yorita et al. [46]	2017	1	86	M		Stomach	Yes	Monoclonal (κ)	Rheumatoid arthritis			NS
38		Cengiz Peker et al. [47]	2017	2	39	M		Stomach	Yes	Polyclonal	CG			NS
39					51	F		Stomach	Yes	Polyclonal	CG			NS
40		Trna et al. [48]	2017	1	77	M		Stomach, heart	NS	NS	NS			Follow-up endoscopy with biopsies–without any difference
41		Altindag et al. [16]	2019	11	81	F		Stomach	No	Polyclonal	Multiple myeloma			Histology report revealed increased distribution in RBs in followup endoscopy
43					84	M		Stomach	Yes	Polyclonal	NS			NS
44					64	M		Stomach	Yes	Polyclonal	NS			NS
45					71	M		Stomach	Yes	Polyclonal	NS			NS
46					79	F		Stomach	No	Polyclonal	Gastric polyps			NS
47					77	F		Stomach	Yes	Polyclonal	Adenocarcinoma			NS
48					44	F		Stomach	Yes	Polyclonal	NS			NS
49					72	M		Stomach	No	Polyclonal	NS			NS
50					64	M		Stomach	No	Polyclonal	CP			NS
51					87	F		Stomach	Yes	Polyclonal	NS			NS
52		Qiao et al. [49]	2019	1	28	M		Stomach	No	Polyclonal	HIV+			NS
53		Umakoshi et al. [50]	2020	1	81	F		Stomach(multifocal)	Yes	Polyclonal	Hepatitis c			NS
54		Yalcin et al. [51]	2020	1	55	M		Stomach	Yes	Polyclonal	CG			almost absent RB after radical treatment of HP
55		Peruhova et al. [52]	2020	1	51	M		Stomach	No	Polyclonal	CG,Iron deficiency anemia			Without endoscopic improvement, histology report showeddecreased RB in second followup and almost absent RB in third follow-up
56		Bozhkova et al. [53]	2021	1	60	F		Stomach	Yes	Polyclonal	Malignant gastric stromal tumor			NS
57		Present study		1	50	M		Stomach	Yes	Polyclonal	Irritable bowel syndrome			almost absent RB after radical treatment of HP

##### HP-positive RBG

Thirty-seven cases, accounting for 67% of total RBGs, with a male-to-female ratio of 1.5:1 (22/15). Mott cells exhibited a monoclonal κ-chain immunophenotype in only nine cases. At least one-third of the patients with HP-positive RBG reported regression of gastritis after eradication of HP. Therefore, an aetiological association was considered between HP infection and RBG. Specifically, surface antigens of HP stimulate the endoplasmic reticulum of plasma cells to produce excessive amounts of IGs or lead to under-secretion of the Golgi apparatus through certain pathways, resulting in intracellular aggregation of abnormal IGs to form RBs [54]. Umakoshi et al. [50] reported a case of multifocal RBG with hepatitis C and HP infection, in which a decreasing number of Mott cells was observed from the mucosa to the submucosa in the ESD specimen. The study attributed this phenomenon to the maturation of plasma cells in the immune system of the gastric mucosa and the IgA secretion features. The overall features included the migration of plasma cell precursors into the lamina propria to mature into plasma cells and the secretion of secretory IgA (S-IgA) into the lumen via the gastric mucosa epithelial cells. Therefore, the formation of RBs may be related to a dysregulation in the IgA secretion mechanism in plasma cells.

##### HP-negative RBG

**Fig. 7:**
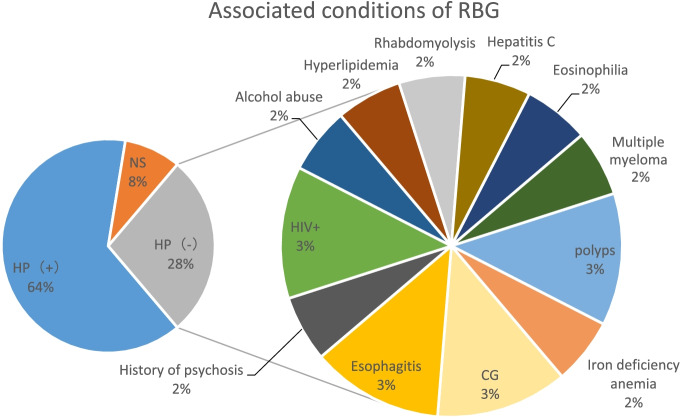
Associated conditions in patients with Helicobacter pylori-negative RBG according to the available literature

Eighteen patients, accounting for 33% of the total cases, with a male-to-female ratio of 1.4:1 (11/7), Mott cells exhibited a monoclonal κ-chain immunophenotype in four cases. HP-negative RBG was first reported in patients with HIV, hepatitis C and multiple osteomyelitis; therefore, it was considered to be associated with abnormalities in the immune function. With an increase in the number of reported cases, HP-negative RBG has also been identified in patients with intestinal tubular adenoma and chronic gastritis (Fig. 7). Given the paucity of the follow-up data, the aetiology and mechanisms of progression of RBG have not yet been described clearly and reliably. Peruhova et al. [52] considered RBG an unstable and dynamic morphological finding that progresses in plasma cell-rich chronic gastritis. Factors contributing to RB formation extend beyond HP infection and may include local degenerative and vascular circulatory phenomena. Some patients with HP-negative RBG demonstrate progressive regression of gastritis with PPI therapy, which may be a viable option for the treatment of the condition.

#### Immunophenotype

RBIDT was divided into polyclonal (positive for both κ and λ chains) and monoclonal (negative for either κ or λ chains) phenotypes based on the expression of the κ and λ chains in Mott cell IGs. The polyclonal phenotype was predominant, whereas the monoclonal phenotype accounted for 25% of the cases (19/76, 16 and three patients with monoclonal κ and λ chains, respectively). Ten patients with the monoclonal phenotype with HP infection had hepatitis C, SLE, rheumatoid arthritis, chronic gastritis and intestinal tubular adenoma without neoplastic lesions. Two of the monoclonal λ chains occurred in the duodenum and sigmoid colon with intestinal polyps.

Traditionally, the polyclonal expression of IGs by B cells was considered one of the indicators to exclude neoplastic lesions, whereas a monoclonal phenotype implied a malignant lesion. There is now a consensus among most scholars that monoclonal hyperplasia of B cells is not a sufficient condition to diagnose malignancy and does not necessarily imply progression to lymphoma, which is considered a focal and non-progressive lesion. In addition, B cells demonstrate monoclonal hyperplasia in certain chronic inflammatory lesions (lymphocytic thyroiditis, chronic hepatitis C, and HP-infected chronic active gastritis) and sicca syndrome and do not subsequently transform into lymphoma. The IG light chain of Mott cells is expressed restrictively in nearly a quarter of cases of RBIDT. Araki et al. [41] stated that it is unreasonable for pathologists to rely solely on the restricted expression of IG light chains in immunohistochemical analysis to diagnose neoplastic lesions. The study reported that the monoclonal hyperplasia of Mott cells in RBG was caused by an inflammatory response, which is consistent with the restricted light chain expression in patients with HP-infected chronic active gastritis. Coyne et al. [37] stated that such a restricted expression is associated with abnormal accumulation of IGs in plasma cells. However, Wolkersdörfer et al. [26] attributed the phenomenon to a mutation in the gene that expresses IGs. Currently, 11 of the 19 cases of monoclonal hyperplasia are from China, 3 from Japan, 2 from the UK and 1 each from the USA, Australia and Switzerland. Considering that the monoclonal phenotypes were commonly identified in China and Japan, Zhang et al. [12] attributed the occurrence to geographical and ethnic differences. In fact, the case in this study is the first Chinese polyclonal phenotype of RBG.

#### Aetiology, pathogenesis and co-morbidities

**Fig. 8:**
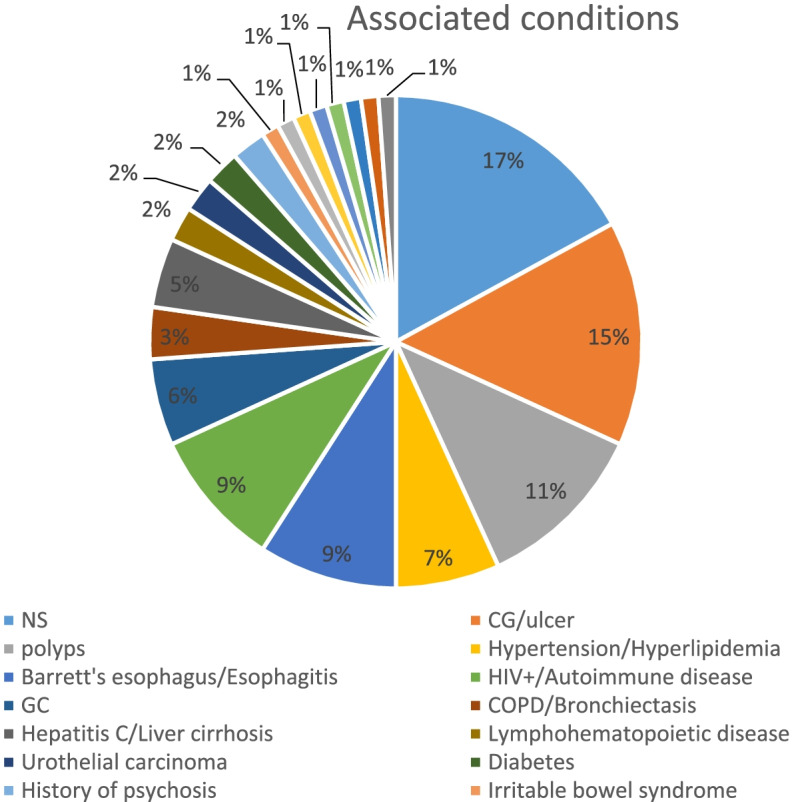
Associated conditions in patients with Russell body inflammation in the digestive tract according to the available literature

The aetiological mechanism of RBIDT remains controversial. Reduction or even regression in the density of RBs has been observed after anti-HP treatment in some patients. In addition, Soltermann et al. [55] identified increased production of RBs in the gastric antrum by HP of the vacA m1 genotype (e.g. in the present case, after 1 week of anti-HP treatment, a gastroscopic biopsy revealed significant regression of RBs in the lamina propria of the mucosa). Based on the aforementioned observations, RBs can be considered to be closely associated with HP infection and irritation. According to statistical data, HP infection is chiefly associated with RBG, whereas patients with RB oesophago-enteritis are hardly infected, and one-third of the patients with RBG are not infected with HP. Therefore, considering HP infection as the cause of all cases of RBIDT is one-sided. Approximately half of all the reported cases of RBIDT (Fig. 8) are accompanied by chronic gastritis and tubular adenomas of the digestive tract, and some patients suffer from peptic ulcers, hypertension, hyperlipidaemia and diabetes mellitus. The common denominator of these diseases is a local vascular inflammatory response and haemodynamic abnormalities. In this regard, HP infection belongs to the category of inflammatory response. However, some patients may have HIV infection, cirrhosis, malignancies (gastric cancer and gastrointestinal stromal sarcomas), immune system-related diseases (rheumatoid arthritis, SLE, among others) and diabetes or may have undergone organ transplantation. The common denominator in the pathology of these conditions is immune dysregulation. Therefore, the aetiology of RBIDT includes an inflammatory response and haemodynamic abnormalities in the local blood vessels or immune dysfunction. The aetiology of the patient in this study was a local inflammatory response caused by irritable bowel syndrome and HP infection.

### Differential diagnosis

Diseases that should be differentiated from RBIDT include SRC of the digestive tract, lymphohaematopoietic tumours accompanied by plasma cell differentiation and extramedullary plasmacytomas.

At low magnification, Mott cells appear similar to mimics signet ring cells, which is associated with a high possibility of misdiagnosis as SRC. However, the nuclei of Mott cells are not allotypic or pathologically divided under high magnification, and the cytoplasm is generally eosinophilic. Bozhkova et al. [56] reported that most AB-PAS are positive in RBs, which is beneficial in their identification. However, PAS is also expressed in SRC; therefore, identifying the two is not helpful. Notably, SRC CK positivity can differentiate between the two conditions. Lymphohaematopoietic tumours accompanied by plasma cell differentiation are identified based on medical history, laboratory examination, histomorphology and specific immunophenotype. Extramedullary plasmacytomas are the most difficult to differentiate from monoclonal hyperplastic RBIDT owing to the lack of early evidence of bone marrow and serological involvement and Bence-Jones albuminuria, which may be accompanied by RB formation. Considering that some chronic inflammatory conditions can demonstrate monoclonal hyperplasia but do not transform into lymphoma at a later stage (including monoclonal expression in one-fourth of the cases of RBIDT), the restricted expression of B-cell IGs is of little significance in the differentiation between the two conditions. Consequently, gastroscopy findings, nuclear atypia and mitotic activity of the pathological cells and biological behaviour should be considered for a comprehensive assessment. Close follow-up is recommended in cases where the nature of the disease cannot be determined.

In the present case, the cytoplasm of the mimics signet ring cells demonstrated mucus-like basophilic rather than eosinophilic nature, which was different from that reported in previous studies. Initially, this phenomenon was hypothesised to be a pseudo-basophilic result of abnormal fixation and handling of the biopsy tissue. However, microscopic observation of the section revealed that the fibrous connective tissue adjacent to the mimics signet ring cells and the red blood cells in the lumen of the microvasculature were eosinophilic, thereby negating this hypothesis. In the present case, PAS and D-PAS special staining were performed to stain the mucogenic granules in the cytoplasm of the surface mucus of the normal mucosal epithelium to a purplish red colour. In this case, the non-staining of mimics signet ring cells indicated the absence of glycogen and neutral mucus in the cytoplasm. In addition, this phenomenon confirmed the basophilic nature of the cytoplasm of the mimics signet ring cells in this case rather than the commonly observed eosinophilic nature in most RBs, which led to the initial misdiagnosis of SRC. However, gastroscopy only revealed white mucosa and mild erosion, and the clinical symptoms were mild, neither of which supported the possibility of malignancy. Therefore, immunohistochemical analysis was performed. Overall, negative CK excluded the possibility of SRC, strong positivity for CD79a and CD138 suggested a plasma cell origin, negative CEA, CK20, ALk and cyclin D1 indicated the absence of other malignancies and Ki-67 (approximately 1% +) suggested a low proliferation index. Three lymphoid follicular dendritic networks were observed around the CD21 mimics signet ring cells, comprising cell clusters with high positive rates of Ki-67, CD10 and Bc1-6 (approximately 30–60%, 10–30% and 40–70%, respectively), indicating the presence of germinal centres and good biological behaviour. Furthermore, there was no restrictive expression of κ or λ, and molecular investigations revealed polyclonal rearrangement of B cells. Malignant lesions such as MALT lymphoma and plasmacytoma accompanied by plasma cell differentiation could be excluded, given that no abnormalities were detected on serology or bone marrow examination. After 1 week of anti-HP treatment, endoscopy performed during the follow-up revealed regression of Mott cells. The patient was followed up closely for 10 months without recurrence. Therefore, this patient was diagnosed with RBG accompanied by polyclonal plasma cell hyperplasia and basophilic RBs.

### Treatment and prognosis

Despite the reported association of autoimmune diseases and EBV-associated gastric cancer with RBIDT, HP infects approximately two-thirds of the patients with RBG, and at least one-third of the patients with HP-positive RBG demonstrate regression of gastritis after eradication of HP. In addition, gastritis has been reported to resolve gradually after PPI treatment in patients with HP-negative RBG. Therefore, it is reasonable to consider it an incidental benign finding. In other words, eradication of the bacteria is essential in patients with HP infection. Moreover, symptomatic treatment with PPI is sufficient for patients without HP infection, and there is no requirement for overtreatment. However, appropriate follow-up and subsequent gastroscopy are essential.

## Conclusions

RBIDT, which often occurs in middle-aged and older men, may present with non-specific gastrointestinal symptoms and endoscopic mucosal changes. The lesions can be seen in almost the entire digestive tract and can be solitary or multiple, most commonly in the stomach, followed by the duodenum, Barrett’s oesophagus, colorectum and ileum. Therefore, the diagnosis and differential diagnosis should be made in conjunction with clinical history, endoscopic findings, laboratory tests, histological features, reliable immunohistochemical and molecular test results. Eradication of the bacteria is essential in patients with HP infection. Moreover, symptomatic treatment with PPI is sufficient for patients without HP infection, and there is no requirement for overtreatment. However, appropriate follow-up and subsequent gastroscopy are essential.

## Data Availability

All the data regarding the findings are available within the manuscript.

## Abbreviations

RBIDT: Russell body inflammation of digestive tract
RB: Russell body
RBs: Russell body
RBG: Russell body gastritis
RBBE: Russell body Barrett’s esophagus
RBD: russel body duodenitis
RBCR: russel body coloproctitis
SRC: mimics signet ring cell carcinoma
CP: colonic polyps
GP: gastritis polyps
F: female
M: male
NS: not stated
CG: chronic gastritis
MGUS: monoclonal gammopathy of undeter mined significance
EBVAGC: EBV-associated gastric carcinoma
HIV: human immunodeficiency virus
GERD: Gastro-esophageal reflux disease
GC: gastric cancer
